# 2-Ethyl-*N*-[(5-nitro­thio­phen-2-yl)methyl­idene]aniline

**DOI:** 10.1107/S1600536811024615

**Published:** 2011-07-09

**Authors:** Ümit Ceylan, Hasan Tanak, Sümeyye Gümüş, Erbil Ağar

**Affiliations:** a Department of Physics, Faculty of Arts & Science, Ondokuz Mayıs University, TR-55139 Kurupelit-Samsun, Turkey; b Department of Physics, Faculty of Arts & Science, Amasya University, TR-55139 Kurupelit-Samsun, Turkey; c Department of Chemistry, Faculty of Arts & Science, Ondokuz Mayıs University, 55139 Samsun, Turkey

## Abstract

In the title compound, C_13_H_12_N_2_O_2_S, the dihedral angle between the benzene and thio­phene rings is 36.72 (8)°. An inter­molecular C—H⋯π inter­action contributes to the stability of the crystal structure.

## Related literature

For the biological properties of Schiff bases, see: Barton & Ollis (1979 ▶); Layer (1963 ▶); Ingold (1969 ▶); for their industrial properties, see: Taggi *et al.* (2002 ▶) and for their reaction properties, see: Aydoğan *et al.* (2001 ▶). For related structures, see: Ağar *et al.* (2010 ▶); Tanak *et al.* (2010 ▶); Demirtaş *et al.* (2009 ▶).
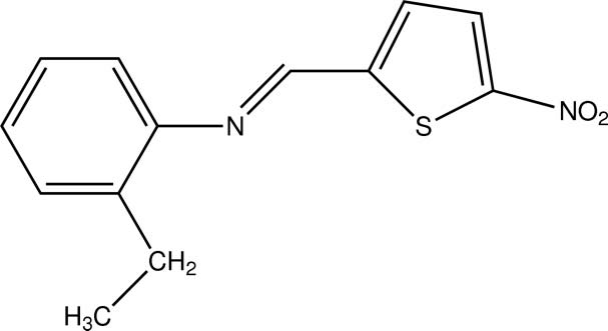


## Experimental

### 

#### Crystal data

C_13_H_12_N_2_O_2_S
*M* *_r_* = 260.31
Monoclinic, 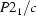

*a* = 11.3578 (4) Å
*b* = 7.4923 (2) Å
*c* = 14.9676 (6) Å
β = 99.589 (3)°
*V* = 1255.89 (7) Å^3^
*Z* = 4
Mo *K*α radiation
μ = 0.25 mm^−1^
*T* = 296 K
0.54 × 0.41 × 0.23 mm

#### Data collection

Stoe IPDS 2 diffractometer
Absorption correction: integration (*X-RED32*; Stoe & Cie, 2002 ▶) *T*_min_ = 0.866, *T*_max_ = 0.954
12190 measured reflections
2468 independent reflections
2195 reflections with *I* > 2σ(*I*)
*R*_int_ = 0.040

#### Refinement

*R*[*F* ^2^ > 2σ(*F* ^2^)] = 0.032
*wR*(*F* ^2^) = 0.087
*S* = 1.05
2468 reflections
176 parameters
H atoms treated by a mixture of independent and constrained refinement
Δρ_max_ = 0.14 e Å^−3^
Δρ_min_ = −0.16 e Å^−3^

### 

Data collection: *X-AREA* (Stoe & Cie, 2002 ▶); cell refinement: *X-AREA*; data reduction: *X-RED32* (Stoe & Cie, 2002 ▶); program(s) used to solve structure: *SHELXS97* (Sheldrick, 2008 ▶); program(s) used to refine structure: *SHELXL97* (Sheldrick, 2008 ▶); molecular graphics: *ORTEP-3 for Windows* (Farrugia, 1997 ▶); software used to prepare material for publication: *WinGX* (Farrugia, 1999 ▶).

## Supplementary Material

Crystal structure: contains datablock(s) I, global. DOI: 10.1107/S1600536811024615/vm2106sup1.cif

Structure factors: contains datablock(s) I. DOI: 10.1107/S1600536811024615/vm2106Isup2.hkl

Supplementary material file. DOI: 10.1107/S1600536811024615/vm2106Isup3.cml

Additional supplementary materials:  crystallographic information; 3D view; checkCIF report

## Figures and Tables

**Table 1:** Hydrogen-bond geometry (Å, °) *Cg*1 is the centroid of the C10–C13/S1 ring.

*D*—H⋯*A*	*D*—H	H⋯*A*	*D*⋯*A*	*D*—H⋯*A*
C7—H8⋯*Cg*1^i^	1.00 (2)	2.94 (2)	3.678 (2)	131.0 (15)

Symmetry code: (i) 
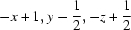
.

**Fig. 1.**
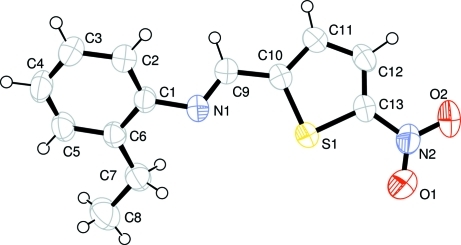
The molecular structure of the title compound, showing the atom-numbering scheme and 50% probability diplacement ellipsoids.

## supplementary crystallographic information

### Comment

Schiff bases, *i.e.*, compounds having a double C=N bond, are used as starting materials in the synthesis of important drugs, such as antibiotics and antiallergic, antiphlogistic, and antitumor substances (Barton *et al.*, 1979; Layer, 1963; Ingold 1969). On the industrial scale, they have a wide range of applications, such as dyes and pigments (Taggi *et al.*, 2002). Schiff bases have also been employed as ligands for the complexation of metal ions (Aydoğan *et al.*, 2001).

We report here the crystal structure of the title new Schiff base compound, (I). The molecular structure is not planar (Fig. 1); the dihedral angle between the C1—C6 benzene and the C10—C13/S1 nitrothiophene ring is 36.72 (8)°. The dihedral angle between the thiophene and nitro group is 3.55 (13)°. The length of the C9=N1 double bond is 1.2694 (18) Å, slightly shorter than standard 1.28 Å value of a C=N double bond and consistent with related structures (Ağar *et al.*, 2010; Tanak *et al.*, 2010; Demirtaş *et al.*, 2009).

The crystal structure is stabilized by π···π stacking interaction (*Cg*(1)···*Cg*(2)^i^ = 3.6618 (9) Å) and by an intermolecular C—H···π stacking interaction (C7–H8···*Cg*(1)^i^ = 2.94 (2) Å) [symmetry code (i): 1 - *x*,-1/2 + *y*,1/2 - *z*; *Cg*(1) and *Cg*(2) are the centroids of rings C10—C13/S1 and C1—C6, respectively).

### Experimental

The compound 2-[(2-ethylphenylimino)methyl]-5-nitrothiophene was prepared by reflux a mixture of a solution containing 5-nitro-2-thiophene-carboxaldehyde (0.025 g 0.160 mmol) in 20 ml ethanol and a solution containing 2-ethylaniline (0.032 g 0.160 mmol) in 20 ml ethanol. The reaction mixture was stirred for 1 h under reflux. The crystals of 2-[(2-ethylphenylimino)methyl]-5-nitrothiophene suitable for X-ray analysis were obtained from ethanol by slow evaporation (yield % 64; m.p 112–114 °C).

### Refinement

C-bound H atoms were positioned geometrically and refined using a riding model, with C—H = 0.93–0.96 Å and *U*_iso_(H) = 1.2–1.5*U*_eq_(C). The position of the H7, H8 and H9 atoms were obtained from a difference map of the electron density in the unit-cell and was refined freely.

### Crystal data

**Table:**

C_13_H_12_N_2_O_2_S	*F*(000) = 544
*M**_r_* = 260.31	*D*_x_ = 1.377 Mg m^−^^3^
Monoclinic, *P*2_1_/*c*	Melting point = 385–387 K
Hall symbol: -P 2ybc	Mo *K*α radiation, λ = 0.71073 Å
*a* = 11.3578 (4) Å	Cell parameters from 18861 reflections
*b* = 7.4923 (2) Å	θ = 1.8–28.0°
*c* = 14.9676 (6) Å	µ = 0.25 mm^−^^1^
β = 99.589 (3)°	*T* = 296 K
*V* = 1255.89 (7) Å^3^	Prism, yellow
*Z* = 4	0.54 × 0.41 × 0.23 mm

### Data collection

**Table:**

Stoe IPDS 2 diffractometer	2468 independent reflections
Radiation source: fine-focus sealed tube	2195 reflections with *I* > 2σ(*I*)
graphite	*R*_int_ = 0.040
Detector resolution: 6.67 pixels mm^-1^	θ_max_ = 26.0°, θ_min_ = 1.8°
rotation method scans	*h* = −13→13
Absorption correction: integration (*X-RED32*; Stoe & Cie, 2002)	*k* = −9→9
*T*_min_ = 0.866, *T*_max_ = 0.954	*l* = −18→18
12190 measured reflections	

### Refinement

**Table:**

Refinement on *F*^2^	Secondary atom site location: difference Fourier map
Least-squares matrix: full	Hydrogen site location: inferred from neighbouring sites
*R*[*F*^2^ > 2σ(*F*^2^)] = 0.032	H atoms treated by a mixture of independent and constrained refinement
*wR*(*F*^2^) = 0.087	*w* = 1/[σ^2^(*F*_o_^2^) + (0.0438*P*)^2^ + 0.2213*P*] where *P* = (*F*_o_^2^ + 2*F*_c_^2^)/3
*S* = 1.05	(Δ/σ)_max_ < 0.001
2468 reflections	Δρ_max_ = 0.14 e Å^−^^3^
176 parameters	Δρ_min_ = −0.16 e Å^−^^3^
0 restraints	Extinction correction: *SHELXL97* (Sheldrick, 2008), Fc^*^=kFc[1+0.001xFc^2^λ^3^/sin(2θ)]^-1/4^
Primary atom site location: structure-invariant direct methods	Extinction coefficient: 0.0197 (19)

### Special details

**Table:**

Experimental. 256 frames, detector distance = 100 mm
Geometry. All e.s.d.'s (except the e.s.d. in the dihedral angle between two l.s. planes) are estimated using the full covariance matrix. The cell e.s.d.'s are taken into account individually in the estimation of e.s.d.'s in distances, angles and torsion angles; correlations between e.s.d.'s in cell parameters are only used when they are defined by crystal symmetry. An approximate (isotropic) treatment of cell e.s.d.'s is used for estimating e.s.d.'s involving l.s. planes.
Refinement. Refinement of *F*^2^ against ALL reflections. The weighted *R*-factor *wR* and goodness of fit *S* are based on *F*^2^, conventional *R*-factors *R* are based on *F*, with *F* set to zero for negative *F*^2^. The threshold expression of *F*^2^ > σ(*F*^2^) is used only for calculating *R*-factors(gt) *etc*. and is not relevant to the choice of reflections for refinement. *R*-factors based on *F*^2^ are statistically about twice as large as those based on *F*, and *R*- factors based on ALL data will be even larger.

### Fractional atomic coordinates and isotropic or equivalent isotropic displacement parameters (Å^2^)

**Table:**

	*x*	*y*	*z*	*U*_iso_*/*U*_eq_	
C13	0.75353 (12)	0.1347 (2)	0.42892 (10)	0.0489 (3)	
H9	0.5748 (13)	0.056 (2)	0.1442 (11)	0.051 (4)*	
H8	0.2532 (19)	−0.043 (3)	0.2366 (14)	0.085 (6)*	
H7	0.243 (2)	0.157 (3)	0.2581 (16)	0.096 (7)*	
S1	0.60815 (3)	0.17034 (5)	0.38059 (2)	0.05021 (14)	
N1	0.44485 (10)	0.15763 (16)	0.20080 (8)	0.0464 (3)	
C1	0.36158 (12)	0.16196 (19)	0.11890 (9)	0.0457 (3)	
C5	0.16094 (14)	0.1298 (2)	0.04281 (11)	0.0600 (4)	
H5	0.0815	0.1006	0.0433	0.072*	
C11	0.75953 (12)	0.0594 (2)	0.28326 (10)	0.0534 (4)	
H11	0.7928	0.0215	0.2338	0.064*	
C2	0.39465 (14)	0.2164 (2)	0.03818 (10)	0.0567 (4)	
H2	0.4738	0.2464	0.0368	0.068*	
C6	0.24197 (12)	0.11916 (19)	0.12317 (10)	0.0482 (3)	
N2	0.78998 (12)	0.1655 (2)	0.52375 (9)	0.0621 (4)	
O2	0.89564 (11)	0.1458 (2)	0.55569 (9)	0.0861 (4)	
C10	0.64167 (12)	0.10510 (19)	0.27810 (9)	0.0453 (3)	
C12	0.82434 (12)	0.0758 (2)	0.37105 (11)	0.0550 (4)	
H12	0.9051	0.0496	0.3873	0.066*	
C4	0.19440 (16)	0.1820 (3)	−0.03736 (11)	0.0686 (5)	
H4	0.1378	0.1873	−0.0899	0.082*	
C7	0.20824 (14)	0.0661 (3)	0.21248 (12)	0.0601 (4)	
O1	0.71418 (13)	0.2089 (2)	0.56818 (9)	0.0905 (5)	
C3	0.31134 (16)	0.2266 (3)	−0.04023 (11)	0.0659 (4)	
H3	0.3339	0.2632	−0.0943	0.079*	
C8	0.07793 (16)	0.0419 (4)	0.21480 (16)	0.0947 (7)	
H8A	0.0672	0.0083	0.2748	0.142*	
H8B	0.0465	−0.0500	0.1728	0.142*	
H8C	0.0366	0.1518	0.1982	0.142*	
C9	0.55012 (12)	0.10376 (19)	0.19795 (10)	0.0472 (3)	

### Atomic displacement parameters (Å^2^)

**Table:**

	*U*^11^	*U*^22^	*U*^33^	*U*^12^	*U*^13^	*U*^23^
C13	0.0404 (7)	0.0530 (8)	0.0504 (8)	0.0000 (6)	−0.0013 (6)	0.0022 (6)
S1	0.0381 (2)	0.0632 (3)	0.0480 (2)	0.00633 (15)	0.00307 (13)	0.00008 (16)
N1	0.0417 (6)	0.0505 (7)	0.0444 (6)	−0.0007 (5)	−0.0007 (5)	0.0011 (5)
C1	0.0440 (7)	0.0468 (7)	0.0433 (7)	0.0034 (6)	−0.0012 (5)	−0.0047 (6)
C5	0.0461 (8)	0.0682 (10)	0.0608 (9)	0.0012 (7)	−0.0058 (7)	−0.0117 (8)
C11	0.0429 (7)	0.0630 (9)	0.0541 (8)	0.0059 (7)	0.0078 (6)	0.0004 (7)
C2	0.0510 (8)	0.0697 (10)	0.0476 (8)	0.0040 (7)	0.0034 (6)	0.0004 (7)
C6	0.0449 (7)	0.0463 (7)	0.0507 (8)	0.0035 (6)	0.0003 (6)	−0.0065 (6)
N2	0.0560 (8)	0.0724 (9)	0.0530 (7)	0.0014 (6)	−0.0050 (6)	−0.0027 (6)
O2	0.0582 (7)	0.1187 (11)	0.0706 (8)	−0.0005 (7)	−0.0210 (6)	−0.0045 (8)
C10	0.0404 (7)	0.0454 (7)	0.0488 (7)	0.0006 (5)	0.0036 (5)	0.0028 (6)
C12	0.0369 (7)	0.0635 (9)	0.0625 (9)	0.0047 (6)	0.0023 (6)	0.0036 (7)
C4	0.0644 (10)	0.0853 (12)	0.0482 (8)	0.0101 (8)	−0.0137 (7)	−0.0109 (8)
C7	0.0494 (8)	0.0670 (10)	0.0630 (9)	0.0006 (7)	0.0066 (7)	0.0042 (8)
O1	0.0784 (9)	0.1339 (13)	0.0568 (7)	0.0181 (8)	0.0037 (6)	−0.0176 (8)
C3	0.0673 (10)	0.0848 (12)	0.0429 (8)	0.0100 (9)	0.0014 (7)	0.0000 (8)
C8	0.0552 (10)	0.140 (2)	0.0904 (14)	−0.0003 (12)	0.0176 (10)	0.0205 (14)
C9	0.0457 (7)	0.0493 (8)	0.0451 (7)	0.0005 (6)	0.0034 (6)	0.0006 (6)

### Geometric parameters (Å, °)

**Table:**

C13—C12	1.351 (2)	C6—C7	1.504 (2)
C13—N2	1.430 (2)	N2—O1	1.2164 (19)
C13—S1	1.7097 (14)	N2—O2	1.2245 (17)
S1—C10	1.7122 (15)	C10—C9	1.4508 (19)
N1—C9	1.2694 (18)	C12—H12	0.9300
N1—C1	1.4185 (17)	C4—C3	1.377 (3)
C1—C2	1.385 (2)	C4—H4	0.9300
C1—C6	1.4077 (19)	C7—C8	1.497 (2)
C5—C4	1.375 (3)	C7—H8	1.00 (2)
C5—C6	1.390 (2)	C7—H7	1.00 (2)
C5—H5	0.9300	C3—H3	0.9300
C11—C10	1.3714 (19)	C8—H8A	0.9600
C11—C12	1.400 (2)	C8—H8B	0.9600
C11—H11	0.9300	C8—H8C	0.9600
C2—C3	1.382 (2)	C9—H9	0.963 (16)
C2—H2	0.9300		
			
C12—C13—N2	125.78 (13)	C9—C10—S1	120.47 (10)
C12—C13—S1	114.58 (11)	C13—C12—C11	110.75 (13)
N2—C13—S1	119.63 (11)	C13—C12—H12	124.6
C13—S1—C10	89.50 (7)	C11—C12—H12	124.6
C9—N1—C1	118.32 (12)	C5—C4—C3	120.31 (14)
C2—C1—C6	120.72 (13)	C5—C4—H4	119.8
C2—C1—N1	121.47 (13)	C3—C4—H4	119.8
C6—C1—N1	117.71 (12)	C8—C7—C6	116.85 (15)
C4—C5—C6	122.17 (15)	C8—C7—H8	109.9 (12)
C4—C5—H5	118.9	C6—C7—H8	110.3 (12)
C6—C5—H5	118.9	C8—C7—H7	110.3 (13)
C10—C11—C12	112.74 (14)	C6—C7—H7	107.2 (13)
C10—C11—H11	123.6	H8—C7—H7	101.0 (17)
C12—C11—H11	123.6	C4—C3—C2	119.19 (16)
C3—C2—C1	120.70 (15)	C4—C3—H3	120.4
C3—C2—H2	119.7	C2—C3—H3	120.4
C1—C2—H2	119.7	C7—C8—H8A	109.5
C5—C6—C1	116.91 (14)	C7—C8—H8B	109.5
C5—C6—C7	123.67 (14)	H8A—C8—H8B	109.5
C1—C6—C7	119.42 (13)	C7—C8—H8C	109.5
O1—N2—O2	123.75 (15)	H8A—C8—H8C	109.5
O1—N2—C13	118.15 (13)	H8B—C8—H8C	109.5
O2—N2—C13	118.10 (14)	N1—C9—C10	121.28 (14)
C11—C10—C9	127.10 (14)	N1—C9—H9	123.5 (9)
C11—C10—S1	112.43 (11)	C10—C9—H9	115.2 (9)
			
C12—C13—S1—C10	−0.24 (12)	C12—C11—C10—C9	179.87 (14)
N2—C13—S1—C10	−179.44 (13)	C12—C11—C10—S1	0.35 (17)
C9—N1—C1—C2	−39.8 (2)	C13—S1—C10—C11	−0.07 (12)
C9—N1—C1—C6	143.95 (14)	C13—S1—C10—C9	−179.63 (12)
C6—C1—C2—C3	−1.2 (2)	N2—C13—C12—C11	179.62 (14)
N1—C1—C2—C3	−177.40 (14)	S1—C13—C12—C11	0.47 (18)
C4—C5—C6—C1	−0.9 (2)	C10—C11—C12—C13	−0.5 (2)
C4—C5—C6—C7	178.76 (16)	C6—C5—C4—C3	−0.2 (3)
C2—C1—C6—C5	1.6 (2)	C5—C6—C7—C8	−6.3 (3)
N1—C1—C6—C5	177.94 (13)	C1—C6—C7—C8	173.42 (17)
C2—C1—C6—C7	−178.07 (15)	C5—C4—C3—C2	0.7 (3)
N1—C1—C6—C7	−1.8 (2)	C1—C2—C3—C4	0.0 (3)
C12—C13—N2—O1	−175.78 (17)	C1—N1—C9—C10	176.56 (12)
S1—C13—N2—O1	3.3 (2)	C11—C10—C9—N1	−176.47 (14)
C12—C13—N2—O2	3.8 (2)	S1—C10—C9—N1	3.0 (2)
S1—C13—N2—O2	−177.09 (13)		

### Hydrogen-bond geometry (Å, °)

**Table:**

Cg1 is the centroid of the C10–C13/S1 ring.

**Table:**

*D*—H···*A*	*D*—H	H···*A*	*D*···*A*	*D*—H···*A*
C7—H8···Cg1^i^	1.00 (2)	2.94 (2)	3.678 (2)	131.0 (15)

Symmetry codes: (i) −*x*+1, *y*−1/2, −*z*+1/2.
